# Level and variation on quality of care in China: a cross-sectional study for the acute myocardial infarction patients in tertiary hospitals in Beijing

**DOI:** 10.1186/s12913-019-3872-0

**Published:** 2019-01-18

**Authors:** Yuqi Zhou, Xi Yao, Guofeng Liu, Weiyan Jian, Winnie Yip

**Affiliations:** 1 School of Public Health, Peking University Health Science Center, Beijing, China; 2 Center for Health Policy and Technology Evaluation, Peking University Health Science Center, Beijing, China; 3 Harvard Global Health Institute, Boston, USA; 4 Harvard T.H. Chan School of Public Health, Beijing, China

**Keywords:** Acute myocardial infarction, Quality of care, Tertiary hospitals, China

## Abstract

**Background:**

Quality of care (QoC) attracts global concerns when unsafe and misuse of healthcare wastes resources and endangers people’s health, especially in low- and middle-income countries. However, little is known about quality of care delivered in China. This study was intended to gauge the quality of care for acute myocardial infarction (AMI) patients in Beijing and identify the quality gaps across tertiary hospitals.

**Methods:**

One thousand two hundred twenty eight patients, covered by Employee Essential Health Insurance Scheme and diagnosed of AMI, was sampled from 14 large comprehensive hospitals in Beijing, China. Chart review study was conducted through the discharge data and medical records of inpatients to evaluate 6 quality outcomes of interest, including the use of aspirin, beta blocker, and statin at discharge; use of aspirin within 24 h at arrival; angiotensin-converting enzyme inhibitors (ACEI) or angiotensin receptor blocker (ARB) for left ventricular systolic dysfunction (LVSD); percutaneous transluminal coronary intervention (PCI) within 90 min at arrival.

**Results:**

Of the 1228 subjects, the mean age was 60.8 (11.8 SD) years and 83.0% were male. The overall medication prescribed was highly compliant with the clinical guidelines (97.0% [95% CI 96.8–97.2] for aspirin and 96.3% [95% CI 96.0–96.5] for statin), except for beta-blocker (83.6% [95% CI 83.0–84.1]) and ACEI/ARB use (61.4% [95% CI 60.7–62.2]). More than half of eligible patients did not receive appropriate PCI therapy (44.0% [95% CI 42.5–45.4]). Great variations across hospitals was observed in aspirin within 24 h and beta-blocker at discharge (*P* < 0.001), and the risk-adjusted results remained robust.

**Conclusion:**

Underuse of recommended treatment and significant variations of quality were found for AMI patients across tertiary hospitals in Beijing. It raised great concerns on poorer quality of care in other less-developed areas with less medical resources. Practical actions are needed in reducing quality gaps to ensure the delivery of quality care.

## Background

The whole world embraces a target to improve the quality of care (QoC) in health systems [1, 2]. A 2013 study funded by the World Health Organization found that a small number of adverse events lead to 43 million injuries a year and the loss of 23 million disability-adjusted life years (DALYs) [3]. Low and middle income countries (LMICs) suffer disproportionate amount of that unsafe medical care, with more than 50% more adverse events and approximately two-thirds of the corresponding loss of DALYs occurring in these countries [4]. However, there is very little empirical evidence on the quality of care delivered in LMICs, including China.

In China, there are around 3000 million outpatient visits and over 150 million inpatient admissions every year [5]. In the last decade, China has tripled its spending on health, with the goal of providing affordable and equitable “quality” basic health care for all by 2020. However, due to a lack of dependable results of QoC measurement [6], the health administration of China has not taken further practice to promote QoC and QoC equity.

There is a growing body of studies in China focusing on the quality of healthcare outcomes such as in-hospital mortality and readmission rate [7, 8]. In March 2016, the China National Report on Health Services and Quality Safety was published [9], signaling the China National Health Commission’s (Ministry of Health) commitment to hospital quality, but the report primarily reported crude mortality and re-admission rates for a small sample of tertiary hospitals [10]. Still there are quite a limited number of researches on the quality of “process” measures in Chinese health institutions [11, 12], extensive gaps undiscovered in quality may impede government effort to better health for all.

Acute myocardial infarction (AMI), as one of the leading mortality causes in China, attracts increasing attention from both researchers and policymakers. In 2013, it was estimated that over 1,394,366 Chinese died of ischemic heart disease [13], which caused great harm to patients’ family and the whole society. Concerns on quality improvement of medical care for AMI patients became an overarching problems. The clinical guidance of AMI was made by Chinese Medical Association (CMA) in 2010 [14], which was based on the latest international evidence and kept consistent with the guidance in developed countries like the European Society of Cardiology (2007), American College of cardiology (2007) and American Heart Association (2009) [15–17]. This makes it reasonable for us to measure the QoC of AMI in Chinese health institutions using the international measurements.

To fill the gap that limited evidence showed the quality of care in China, this study was designed to gauge the level and variation of QoC for AMI patients in 14 tertiary hospitals in Beijing. As the capital city of China, Beijing ranks top in economic and social development with the urbanization rate of over 80%, which is also the center of health resources and can represent the first-class medical services of high-quality across the country. All the 14 hospitals are general hospitals equipped with over 1000 beds each, and enjoy a good reputation as “good hospitals” among Chinese patients.

## Methods

### Study design

For the tertiary general hospitals in urban Beijing, we selected the top 14 hospitals with more than 1000 beds and over 200 AMI inpatients annually, excluding military hospitals. From the patients covered by Beijing Employee Essential Health Insurance Scheme (BEHI), inpatients diagnosed of AMI and received percutaneous coronary intervention (PCI) or coronary artery bypass grafting (CABG) during hospitalization were chosen. Given the limited budget, we randomly selected 30% from AMI inpatients at each hospital and yielded a sample of 1228 cases in 2014 (see in Appendix Table 4).

### Data collection

Chart reviews of patients’ records were conducted to collect discharge data and medical record. Discharge data includes information on patients’ ID, age, gender, principal and secondary diagnosis, and date of admission and discharge. Information on patients’ medication (like aspirin, beta-blocker, ACEI, AR and statin) during hospitalization and treatment procedure (like PCI) was obtained through medical record review, and was utilized to compute QoC indicators of AMI. Data extraction chart related to the targeted QoC indicators was developed. We invited and trained 10 professional clinical doctors with over ten-year working experience as reviewers, to collect information from medical records for further data analyses.

### QoC indicators

Consistent with the clinical guideline recommendations of Chinese Medical Association, the QoC measures from National Quality Forum (NQF) of the US were used to evaluate 6 quality outcomes of interest [18], specifically including:

1. Aspirin at arrival: Percentage of AMI patients without aspirin contraindications who received aspirin within 24 h after hospital arrival.
2. Aspirin prescribed at discharge: Percentage of AMI patients without aspirin contraindications who are prescribed aspirin at hospital discharge.
3. Beta-blocker prescribed at discharge: Percentage of AMI patients without beta blocker contraindications who are prescribed a beta blocker at hospital discharge.
4. Statin prescribed at discharge: Percentage of AMI patients with low density lipoprotein (LDL) no less than 100 mg/dL, or on cholesterol reducing therapy prior to hospitalization who are discharged on a statin medication.
5. ACEI or ARB for left ventricular systolic dysfunction (LVSD): Percentage of AMI patients with LVSD and without both angiotensin converting enzyme inhibitor (ACEI) and angiotensin receptor blocker (ARB) contraindications who are prescribed an ACEI or ARB at hospital discharge.
6. PCI received within 90 min of hospital arrival: Percentage of AMI patients receiving PCI therapy during the hospital stay with a time from hospital arrival to PCI of 90 min or less.

We didn’t use the indicator of Fibrinolytic therapy received within 30 min of hospital arrival in our study, for only 15 cases in our sample received it and 5 out of the 14 hospitals didn’t use Fibrinolytic therapy.

### Statistical analysis

Both the quality measures of the entire sample and each hospital were observed by calculating the proportion of patients who received the guided treatment. Analysis of Variance reporting *P* values tested the differences of quality between 14 hospitals. The linear probability model was used to develop comparable quality outcomes, adjusted for patient demographic information and severity of comorbidity scored by Charlson Comorbidity Index (CCI) [19, 20]. All data analysis was conducted with STATA software (version 14.0).

## Results

Of the 1228 AMI patients (shown in Table 1), nearly half (48.2%) were aged between 40 and 60, followed by those between 60 and 80 (43.3%). The younger and high older suffering from AMI were both less than 5%. Most were male, 1014 (85.0%) were diagnosed with ST-elevation myocardial infarction, and 1187 (96.7%) were treated with percutaneous coronary intervention. As for the severity of comorbidity, 76.8% had the CCI no more than 1, only 1.9% larger than 3, and approximate one fifth with the CCI between 1 and 3. The mean (SD) expenditure per capita and length of stay were CN¥ 73,702.44 (32,293.72) and 11.08 (6.91) respectively.

**Table 1:** Patient characteristics for all hospitals

Variables		*N* (%)
N		1228
Age	0–18	0 (0)
	19–40	48 (3.9)
	41–60	592 (48.2)
	61–80	532 (43.3)
	> 80	56 (4.6)
Gender	Male	1019 (83.0)
	Female	209 (17.0)
Comorbidity	CCI ≤ 1	943 (76.8)
	1 < CCI ≤ 2	193 (15.7)
	2 < CCI ≤ 3	69 (5.6)
	CCI > 3	23 (1.9)
AMI	STEMI	1014 (85.0)
	NSTEMI	179 (15.0)
Therapy	PCI	1187 (96.7)
	CABG	40 (3.3)
Expenditure per admission (China Yuan)	Mean and SD	73,702.44 (32,293.75)
Length of stay (days)	Mean and SD	11.08 (6.91)

Note: *CCI* = Charlson Comorbidity Index, *AMI* = acute myocardial infarction, *STEMI* = ST-Elevation Myocardial Infarction, *NSTEMI* = Non-STEMI, *PCI* = percutaneous coronary intervention, *CABG* = coronary artery bypass grafting

Outcomes of each quality measure for the entire sample were reported in Table 2. Aspirin within 24 h at admission was performed in 94.38% of AMI patients. Eligible patients without corresponding contraindications who received aspirin, beta-blocker and statin prescribed at discharge were 91.11, 83.75, and 96.43%, respectively. All the indicators for medication showed a high level of quality in the sample hospitals, and over 80% were treated with recommended care, even for beta-blocker, the lowest index among them. However, there were considerable variations between hospitals, especially for aspirin at arrival and beta-blocker at discharge (*P* < 0.001).

**Table 2:** Level of QoC for all hospitals

Measures	No. of cases with complete information	Recommended care delivered or adverse outcome *N* (%)	No. of missing data	*P* value*
Aspirin at arrival	1139	1075 (94.38)	89	<0.001
Aspirin prescribed at discharge	1176	1142 (91.11)	52	0.577
Beta-blocker prescribed at discharge	1175	982 (83.75)	53	<0.001
Statin prescribed at discharge	1176	1134 (96.43)	52	0.070
ACEI or ARB for LVSD	54	34 (62.96)	N/A	N/A
PCI received within 90 min of hospital arrival	357	171 (47.90)	N/A	<0.001

*ACEI* = angiotensin-converting enzyme inhibitors, *ARB* = angiotensin receptor blockers, *LVSD* = left ventricular systolic dysfunction, *PCI* = percutaneous coronary intervention, *N/A* = not applicable

*P value tests the hypothesis that there are no differences in quality of care between the 14 hospitals

For the indicator “ACEI or ARB for LVSD”, 54 cases out of the total sample were diagnosed of LVSD and had neither ACEI nor ARB contraindications, among whom 34 cases (62.96%) were prescribed ACEI or ARB.

Overall, 1187 patients received PCI, in which 243 cases had no time record for the initiation of PCI procedure. In the 985 cases with time record, only 357 cases’ records were stored down-to-minute, while the rest merely contained the specific date. Based on the information of time record, we found 171 out of the 357 cases (47.90%) received PCI within 90 min of hospital arrival.

Risk-adjusted rates for the entire sample were displayed in Table 3. All the measures were lower and had a smaller dispersion after controlling random variations due to different patient case mix. Similar with the crude rates, four medication indexes indicated the high compliance of clinical guidelines, while ACEI or ARB for LVSD and PCI within 90 min were greatly underused.

**Table 3:** Contrast of the quality measures between crude and risk-adjusted rates

Measures	Crude rate (%) [SD]	Risk-adjusted rate (%) [95% CI]^a^
Aspirin at arrival	94.38 [0.230]	94.17 [93.88–94.45]
Aspirin prescribed at discharge	97.11 [0.168]	96.98 [96.75–97.22]
Beta-blocker prescribed at discharge	83.57 [0.371]	83.55 [83.01–84.11]
Statin prescribed at discharge	96.42 [0.186]	96.28 [96.03–96.53]
ACEI or ARB for LVSD	62.96 [0.487]	61.41 [60.65–62.17]
PCI received within 90 min of hospital arrival	47.90 [0.500]	43.96 [42.54–45.38]

Note: *ACEI* = angiotensin-converting enzyme inhibitors, *ARB* = angiotensin receptor blockers, *LVSD* = left ventricular systolic dysfunction, *PCI* = percutaneous coronary intervention

^a^Age, gender, therapy and comorbidities (CCI) were controlled

**Fig. 1:**
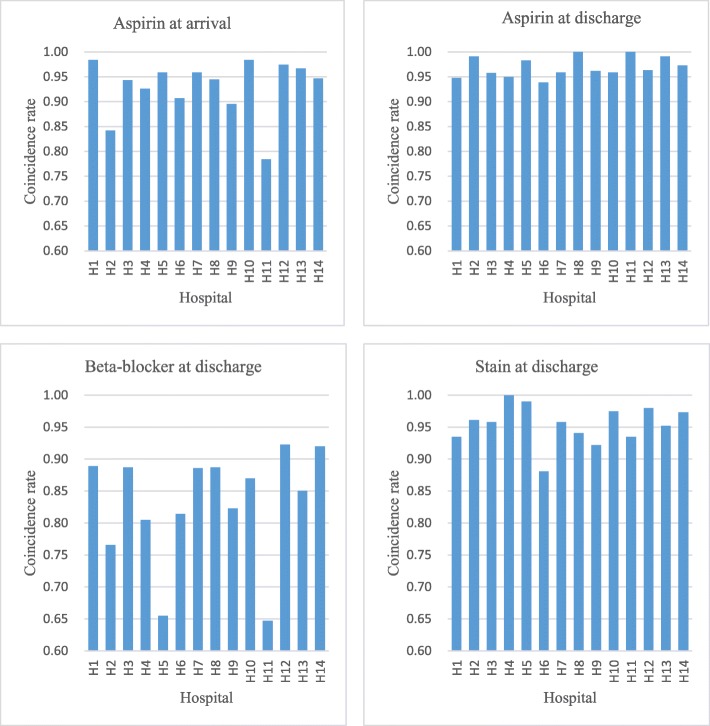
Compliance of quality measures for each hospital. Note: age, gender, therapy and comorbidities (CCI) were controlled

Quality measures for each hospital were shown in Fig. 1 (see more in Appendix Table 5). Pronounced variations were noted as aspirin at arrival and beta-blocker at discharge varying from 78.4% (95% CI: 78.0–78.8) to 98.4% (95% CI: 98.2–98.6) and 64.7% (95% CI: 63.4–65.9) to 92.3% (95% CI: 91.7–93.0), respectively. Over 96.0% patients received aspirin within 24 h in the highest quartile of hospitals, while the lowest quartile delivered aspirin to less than 90%. Around 92% obtained beta-blocker at discharge in the highest two hospitals, but only 65% in the lowest two hospitals. This inconsistence across hospitals suggested the quality gap in medication. Aspirin and statin at discharge were prescribe for more than 95% patients in most hospitals, but there was room for improvement since the best hospitals has already delivered recommended care for all patients.

## Discussion

This study assessed the quality of care on AMI patients in tertiary hospitals in Beijing with a set of commonly-used international measures. Our results also indicated the variations of quality between these hospitals. Most hospitals performed well in the prescription of aspirin and statin at discharge, but substantial inter-hospital variations exist in aspirin at arrival and beta-blocker at discharge. Although only a small number of patients were diagnosed as left ventricular systolic dysfunction, the use of ACEI/ARB was rather disappointing with around 40% of patients untreated. The low rate of PCI within 90 min radiated the signal of inappropriate medical treatment, but comparison between hospitals should consider the bias incurred by incomplete time information of PCI operation for some patients.

Compared to the results of the first national representative study, China Patient-centered Evaluative Assessment of Cardiac Events Retrospective Study of Acute Myocardial Infarction (China PEACE) in 2011 [21], great achievement was made in terms of these quality measures (see in Appendix Table 6). The use of aspirin at arrival and statin at discharge increased slightly from 91.2% (95% CI: 90.5–91.8) to 94.2% (95% CI: 93.9–94.5) and 92.5% (95% CI: 91.9–93.1) to 96.3% (95% CI: 96.0–96.5) respectively, while ACEI/ARB was slightly lower than that of China PEACE [22–25]. It was worth noting that the similar decreasing trend of ACEI/ARB usage, 70.7% (95% CI: 69.2–72.2) in 2006 to 66.4% (95% CI: 65.2–67.5) in 2011, was found in China PEACE. As for beta-blocker, we concentrated on the prescription at discharge rather than the use within 24 h in China PEACE. Both our study and China PEACE failed to report the precise rate of PCI therapy within 90 min because of data missing for key information. In addition, this study revealed considerable variations in QoC across hospitals, while China PEACE focused on regional differences. The overall improvement the expected result because these findings came from tertiary hospitals in Beijing, which represents the top level of medical care in China. However, the underuse of ACEI/ARB, beta-blocker and PCI therapy indicates the defect in quality of care, since these treatments are proved effective in improving prognosis and reducing mortality risk. Inconsistence about quality of care delivered in different hospitals entailed monitoring and evaluating the implementation of quality management and clinical guidelines.

Similar with the Cooperative Cardiovascular Project in America two decades ago, substantial variation in quality of AMI was observed and there were ample opportunities for improvement [26–28]. The Medicare Health Care Quality Improvement Program had been focusing on the healthcare of AMI patients since 1992, and enormous effort in the management of AMI ensured the appropriate care for more patients in the US [26]. Our findings also signals the inconsistence on implementing clinical guidelines of AMI in different hospitals. Although there are explicit standards released by Chinese Medical Association and National Health Commission, these hospitals failed to provide evidence-based medical care for all the AMI patients. It can be inferred that patients in other junior hospitals or underdeveloped areas may be putted at higher risk of sufferings because of low-quality healthcare. It is urgent for Chinese government to recognize the insufficient quality and take actions in improve hospital care.

Public hospital is the main provider in healthcare delivery system in China. The government has endeavored to strengthen the capacity of hospitals and increase the access to health services, responding to people’s surging health demand. Improvement in access to healthcare is encouraging, but no evidence shows the delivery of high-quality care to patients. Gaps in quality of care, especially the underuse of recommended necessary therapies, are probably the consequences of multiple factors. First, inadequate knowledge about the potential risk without appropriate treatment. Previous studies has pointed out that physicians are not trained under the standard clinical guidelines and do not insist on providing evidence-based healthcare [21]. Second, lack of scientific management tools on measuring quality of care. The published report of National Health Commission focused on the outcome measures, but neglected the process quality. On the one hand, there is no systematic set of quality indicators about the management of medical process; on the other hand, no complete documented data is available. Thus it is difficult for the health authorities to identify the problems and find specific solutions. Last, insufficient policy incentives. The hospital management and staff promotion should be linked with performance assessment to ensure the continuous impetus of quality improvement, otherwise the clinical guidelines and quality control measures are only unenforceable framework.

This study has several limitations. First, this research was a cross-section study in local settings. Samples were selected from patients of 14 large hospitals in Beijing in a single year. Deliberation is needed to reach a nationwide conclusion, given the limitation of sample representativeness. Besides, time-variant changes are not observed. Although we inferred the quality issues around the country basing on the hypothesis that QoC in tertiary hospitals of good reputation can represent the first-class medical care in China and compared our findings with China-PEACE in 2011, further study should provide more concrete and precise evidence on the changing of quality in health care. Second, our measures of quality were based on the documented medical records of AMI patients. Standard and completeness of the stored information may differ across hospitals and over time. For instance, the smoking cessation indicator was not reflected due to missing records. Third, the difficulty in data collecting. Professional clinical physicians were trained to collect data from medical records in our study, but it will be a great challenge when this method is generalized to other hospitals in China. The cost and feasibility in data acquisition may restrict the nationwide assessment of healthcare quality, unless the establishment of a well-functioned hospital electronic information system with unified standard. QoC in other medical facilities will raise great concern, since there is no comparable quality performance information system.

## Conclusion

This study found significant variations in quality of care for AMI patients across tertiary hospitals in China and contributed to the limited empirical literature of healthcare quality in developing countries. The underuse of evidence-based treatment delivered to eligible patients alerts the government to the emergency of protecting patients’ health outcomes. We firmly believe that quality improvement will save thousands of lives and enhance health conditions of the people, with trained physicians, accurate measurement, scientific monitoring and effective incentives.

## Appendix 1

**Table 4:** Sample cases of each hospitals

The 14 sample hospitals	Bed sizes	Number of AMI discharges covered by BEHI	in which, the cases with PCI or CABG	Sample cases
H1	1500	326	281	88
H2	1287	522	367	107
H3	1609	261	225	71
H4	1420	342	276	87
H5	1871	581	421	131
H6	1503	181	125	36
H7	1448	315	251	83
H8	1037	155	123	37
H9	1006	250	180	57
H10	1162	448	405	126
H11	1032	170	105	32
H12	1147	521	403	124
H13	1256	572	428	131
H14	1300	420	381	118
Total cases	N/A	5064	3791	1228

*BEHI*=Beijing Employee Essential Health Insurance Scheme

Sampling: 30% AMI cases with PCI or CABG from each of the 14 hospitals were randomly selected to yield a sample of 1228 case, which met the sample size requirement for subsequent data analysis

## Appendix 2

**Table 5:** Estimates of Linear Probability Regression Model for the quality measures of each hospital

Hospital	H1	H2	H3	H4	H5	H6	H7	H8	H9	H10	H11	H12	H13	H14
N	88	107	71	87	131	36	83	37	57	126	32	124	131	118
Aspirin within 24 h	0.984 (0.979, 0.989)	0.842 (0.838, 0.847)	0.943 (0.937, 0.948)	0.926 (0.923, 0.928)	0.959 (0.955, 0.964)	0.907 (0.903, 0.911)	0.959 (0.954, 0.965)	0.945 (0.941, 0.949)	0.895 (0.89, 0.899)	0.984 (0.982, 0.986)	0.784 (0.78, 0.788)	0.974 (0.971, 0.978)	0.967 (0.965, 0.97)	0.947 (0.944, 0.949)
Aspirin prescribed at discharge	0.948 (0.935, 0.962)	0.991 (0.983, 0.999)	0.958 (0.95, 0.966)	0.95 (0.947, 0.953)	0.983 (0.975, 0.99)	0.939 (0.929, 0.949)	0.959 (0.951, 0.967)	1 (0.993, 1.008)	0.962 (0.955, 0.968)	0.959 (0.955, 0.963)	1 (0.994, 1.006)	0.963 (0.956, 0.969)	0.991 (0.983, 1)	0.973 (0.968, 0.978)
Beta-blocker prescribed at discharge	0.889 (0.867, 0.91)	0.766 (0.76, 0.771)	0.887 (0.878, 0.897)	0.805 (0.797, 0.812)	0.655 (0.649, 0.661)	0.814 (0.801, 0.826)	0.886 (0.877, 0.895)	0.887 (0.875, 0.9)	0.823 (0.814, 0.832)	0.87 (0.865, 0.876)	0.647 (0.634, 0.659)	0.923 (0.917, 0.93)	0.85 (0.835, 0.864)	0.92 (0.914, 0.926)
Statin prescribed at discharge	0.935 (0.923, 0.948)	0.961 (0.952, 0.971)	0.958 (0.949, 0.966)	1 (0.998, 1.002)	0.99 (0.982, 0.998)	0.881 (0.873, 0.888)	0.958 (0.949, 0.967)	0.941 (0.936, 0.946)	0.922 (0.916, 0.929)	0.975 (0.972, 0.978)	0.935 (0.932, 0.939)	0.98 (0.973, 0.987)	0.952 (0.944, 0.96)	0.973 (0.968, 0.978)
ACEI or ARB for LVSD	0.627 (0.596, 0.659)	0.65 (0.628, 0.672)	0.603 (0.571, 0.634)	0.637 (0.611, 0.663)	0.596 (0.573, 0.619)	0.623 (0.585, 0.66)	0.622 (0.595, 0.648)	0.615 (0.572, 0.657)	0.646 (0.612, 0.68)	0.583 (0.559, 0.607)	0.594 (0.542, 0.646)	0.616 (0.593, 0.64)	0.615 (0.591, 0.639)	0.593 (0.566, 0.62)
PCI received within 90 min of hospital arrival	0.674 (0.66, 0.689)	0.514 (0.504, 0.525)	0.664 (0.649, 0.678)	0.42 (0.408, 0.432)	0.302 (0.292, 0.312)	0.237 (0.215, 0.259)	0.562 (0.55, 0.574)	0(−0.078, −0.039)	0.712 (0.701, 0.723)	0.366 (0.356, 0.376)	0.688 (0.669, 0.706)	0(−0.113, −0.091)	0.585 (0.574, 0.596)	0.65 (0.637, 0.662)

Note: means with 95% confidence interval in parentheses were displayed, after controlling for age, gender, therapy and comorbidities (CCI)

## Appendix 3

**Table 6:** Performance on quality measures in the sample hospitals versus China-PEACE

Quality indicators	China-PEACE^a^		14 Beijing hospitals
	2006 (*n* = 3626)	2011 (*n* = 6643)	2014 (*n* = 1228)
Aspirin within 24 h	86.8 (85.7–87.9)	91.2 (90.5–91.8)	94.2 (93.9–94.5)
Aspirin prescribed at discharge	N/A	N/A	97.0 (96.8–97.2)
Beta-blocker within 24 h	63.7 (61.4–66.0)	57.3 (55.6–59.0)	N/A
Beta-blocker prescribed at discharge	N/A	N/A	83.6 (83.0–84.1)
Statin prescribed at discharge	75.9 (74.5–77.3)	92.5 (91.9–93.1)	96.3 (96.0–96.5)
ACEI or ARB for LVSD	70.7 (69.2–72.2)	66.4 (65.2–67.5)	61.4 (60.7–62.2)

*ACEI* = angiotensin-converting enzyme inhibitors, *ARB* = angiotensin receptor blockers, *LVSD* = left ventricular systolic dysfunction, *N/A* = not applicable

Note: percentage with 95% CI in the parentheses is shown

^a^Data comes from China Patient-centered Evaluative Assessment of Cardiac Events Retrospective Study of Acute Myocardial Infarction (China PEACE)

## Abbreviations

ACEI: angiotensin-converting enzyme inhibitors
AMI: acute myocardial infarction
ARB: angiotensin receptor blocker
LVSD: left ventricular systolic dysfunction
PCI: percutaneous transluminal coronary intervention
QoC: quality of care
